# An activity canyon characterization of the pharmacological topography

**DOI:** 10.1186/s13321-016-0153-3

**Published:** 2016-08-19

**Authors:** Varsha S. Kulkarni, David J. Wild

**Affiliations:** School of Informatics and Computing, Indiana University, Bloomington, IN USA

## Abstract

**Background:**

Highly chemically similar drugs usually possess similar biological activities, but sometimes, small changes in chemistry can result in a large difference in biological effects. Chemically similar drug pairs that show extreme deviations in activity represent *distinctive* drug interactions having important implications. These associations between chemical and biological similarity are studied as discontinuities in activity landscapes. Particularly, activity cliffs are quantified by the drop in similar activity of chemically similar drugs. In this paper, we construct a landscape using a large drug-target network and consider the rises in similarity and variation in activity along the chemical space. Detailed analysis of structure and activity gives a rigorous quantification of distinctive pairs and the probability of their occurrence.

**Results:**

We analyze pairwise similarity (*s*) and variation (*d*) in activity of drugs on proteins. Interactions between drugs are quantified by considering pairwise *s* and *d* weights jointly with corresponding chemical similarity (*c*) weights. Similarity and variation in activity are measured as the number of common and uncommon targets of two drugs respectively. Distinctive interactions occur between drugs having high *c* and above (below) average *d* (*s*). Computation of predicted probability of distinctiveness employs joint probability of *c*, *s* and of *c*, *d* assuming independence of structure and activity. Predictions conform with the observations at different levels of distinctiveness. Results are validated on the data used and another drug ensemble. In the landscape, while *s* and *d* decrease as *c* increases, *d* maintains value more than *s*. *c* ∈ [0.3, 0.64] is the transitional region where rises in *d* are significantly greater than drops in *s*. It is fascinating that distinctive interactions filtered with high *d* and low *s* are different in nature. It is crucial that high *c* interactions are more probable of having above average *d* than *s*. Identification of distinctive interactions is better with high *d* than low *s*. These interactions belong to diverse classes. *d* is greatest between drugs and analogs prepared for treatment of same class of ailments but with different therapeutic specifications. In contrast, analogs having low *s* would treat ailments from distinct classes.

**Conclusions:**

Intermittent spikes in *d* along the axis of *c* represent *canyons* in the activity landscape. This new representation accounts for distinctiveness through relative rises in *s* and *d*. It provides a mathematical basis for predicting the probability of occurrence of distinctiveness. It identifies the drug pairs at varying levels of distinctiveness and non-distinctiveness. The predicted probability formula is validated even if data approximately satisfy the conditions of its construction. Also, the postulated independence of structure and activity is of little significance to the overall assessment. The difference in distinctive interactions obtained by *s* and *d* highlights the importance of studying both of them, and reveals how the choice of measurement can affect the interpretation. The methods in this paper can be used to interpret whether or not drug interactions are distinctive and the probability of their occurrence. Practitioners and researchers can rely on this identification for quantitative modeling and assessment.

## Background

The structural features of a drug compound describe its physicochemical properties that determine its biological activity on protein targets [1–4]. A vast number of combinations or analogs that result from small changes in chemical structure may not contribute to the diversity in biological activity or functionality of drugs. According to the similarity principle, structurally similar drugs tend to possess similar activity profiles, meaning that they would behave similarly on a particular protein [5, 6]. In addition, the impact of subjectivity in the decision making of medicinal chemists is known [7]. Thus the synthesis of new drugs, by introducing modifications in chemical compositions, is a complex process. The clinical phases of drug preparation are known to focus on a few properties [3, 4] but little is known about how the chemical structure is associated with biological activity of drugs. As established previously for drug target data, few drugs are active on many more targets than average while a large number of drugs are active on much fewer targets [8, 9]. This points to the discovery of certain molecular combinations having highly versatile functionalities. On the other hand, if targets are sufficiently distinct in their chemical nature, then the high druggability of few targets points to low specificity of a large number of medicines. Dissimilarity in activities of drugs when measured pairwise, often reveals pairs of drugs with highly dissimilar activity. The pairwise associations or interactions of drugs are quantified with their chemical similarity and similarity or dissimilarity in their activities. Is variation in activity of drugs necessarily based on their chemical structures?

Medicinal chemistry has relied on the similarity principle [3–6]. It implies that two structurally similar medicines tend to have similar (if not identical) activity profiles. However, many exceptions are known. For instance, chemically analogous tricyclic compounds Promethazine, Chlorpromazine, Imipramine possess distinct therapeutics [1, 2]. Highly similar PPAR-G drugs—Rosiglitazone and Troglitazone have very different side-effect and adverse event profiles. Development of medicines and analogs with many targets facilitates the production of multipurpose medicines. It also leads to the emergence of activity cliffs [10–12], specifying pairs of structurally similar drugs having highly variant biological activities. These cliff-like interactions between drugs show extreme behaviors of pairs of chemically similar drugs showing unusually large deviations in activities. They are quantified by the drop in similar activity of two drugs. However, one may use the rise in pairwise variation of their activities instead. Therefore, effectively such interactions between pairs of drugs can be termed as distinctive. The distinctiveness can be measured as the either the drop in similar activity or rise in variant activity of two drugs. In this paper, we develop a quantitative method to predict the probability of finding *distinctive* interactions using these measures and try to interpret them.

Activity landscape of drugs or the pharmacological *topography* is a two-dimensional space of chemical structure and activity to which a pair of drugs can be mapped. In any landscape, the activity cliffs are represented as discontinuities occurring due to distinct drops in similar activity of chemically similar drugs. These are distinctive drug interactions studied with great interest. Here, we analyze them using topographical features of the landscape. Researchers have studied activity cliffs using graphical methods of interpreting the landscape. They have given detailed specifications of cliffs [10–12]. The series of cliffs in the landscape is characterized as activity ridge when many structural analogs possess high activity variation [11, 12] and activity islands are used to specify the structurally similar compounds based on some descriptors [13]. Quantitative analysis of the structure activity relationships is of growing importance to researchers but known to be less effective for studying cliffs formed by analogs having little structural variation [3]. In previous work, quantitative analysis has relied mainly on measures such as—structure activity landscape index (SALI) and some algorithmic or statistical analysis [12]. However, these methods and specifications of cliff (or distinctive) interactions are rather insufficient as they are threshold driven and offer a descriptive analysis of patterns. Moreover, increased activity variation may not indicate decrease in activity similarity particularly when the magnitude of similarity is determined by number of commonly active targets. Previous studies have not differentiated between these two measures of quantifying the aberrations. Further, the magnitude of data considered for the analysis can significantly change the patterns of drug activity on targets and the inferences on similar activity [14].

This paper attempts to provide a mathematical basis of the extreme deviations observed. It would facilitate identification of distinctive interactions on different scales of measurements. The paper introduces a probabilistic analysis of the pharmacological topography and uses two measures of activity (similarity, *s* and variation, *d*) jointly with the corresponding chemical similarity *c* to investigate distinctiveness. The measures are proportional to Jaccard index and represent weighted networks of drugs [15, 16]. The weights of the connections between drugs represent the strengths of their interactions. Pairwise activity similarity, *s* refers specifically to the number of commonly active targets of two drugs. Using probability distributions of *s*, *d*, *c* we identify medicinal categories involved in the highly distinctive interactions. The predicted probabilities of *s* and *d* jointly with *c* are computed. This gives the probability of distinctiveness at varying levels. The level of distinctiveness of a pair of drugs (or drug interaction) is specified according to the magnitudes of the attributes *s*, *d*, *c* for that pair. We find that the choice of the measurement (low *s* or high *d*) affects the interpretation of distinctiveness and the topography. These measures could impact the way in which we interpret the relation between two drugs in terms of their selectivity. Drug selectivity is a property exhibited by a drug when it is active on a protein as opposed to different proteins [3, 4]. Thus *s* and *d* can indicate how commonly and uncommonly selective the pairs of drugs are, but this is not necessary.

Highly distinctive interactions can be filtered as structurally similar drugs having either the most variant or least similar activity profiles. Intriguingly, there is little consensus on the distinctive interactions filtered with the two measures. We study the diversity of pharmacological space using a large network of drugs and protein targets. The similarity principle is contradicted in the region where cliffs are present, however, the sizes of these cliffs are variable [12]. Structural or chemical similarity of two drugs (*c*) corresponds to a divergence of both the nature and size of their activity profiles. Plausibly, a slight change in choice of molecules results in a significantly dissimilar analog of a drug while not altering its functioning on many targets. Conversely, the activity profiles of new medicines may be vastly different from those of their chemical analogs. The propensity of distinctiveness in drug interactions has implications on drug substitution. The discovery of multi-target drugs is beneficial for drug repurposing and it can result from small structural changes, which would affect distinctiveness of pairs of drugs. The method of analyzing distinctiveness is, therefore, important. Through a rigorous quantitative assessment of the activity landscape, this analysis aims to contribute not only to drug design but also to the decision making of the pharmacologists. Practitioners can apply these methods and criteria to classify the drug interactions as distinctive.

In the present pharmacological space, distinctive interactions are more probable than non-distinctive interactions. Non-distinctive interactions are regular as they conform to the similarity principle. Distinguishing among the distinctive interactions requires precision. The range of *d* is much higher than that of *s*. Quantitative comparison of the landscape reveals that structural and biological similarities, two a priori independent manifestations of drug interactions, are unevenly associated and maybe complementary. The presence of distinctiveness is highlighted by both *s* and *d*. As chemical similarity increases, both measures decrease. However, the decrease in *s* is more significant than that in *d*, as *d* maintains its above average value. We show that activity variation is a more suitable measure for characterization of the pharmacological interaction space than activity similarity. Intermittent rises in *d* with chemical similarity maybe interpreted as activity *canyons* or *gorges* of varying levels. In this representation, the rises in both measures can be assessed relative to their respective ranges of magnitudes and compared across all pairs. The rises in activity similarity and variation are interpreted as increase in non-distinctiveness and distinctiveness respectively, characterized as activity canyons instead of cliffs. Therefore, distinctiveness is a general term that may represent abrupt deviations in the landscape as cliffs or canyons depending on the method of measurement. Moreover, the canyon representation allows for the quantitative analysis of ‘how distinctive and how probable?’, demarcating the pharmacological subspace for finding distinctiveness. These rises in activity variation are probable all along the chemical space and more distinct than drops in *s*. The predicted probability model introduced in this paper helps in identifying distinctive interactions and the probability of their occurrence in the landscape. It also helps in identifying distinctive drug associations that are not only significant and rare but also less apparent.

## Results and discussion

### The drug network

We analyzed the bipartite graph of 1354 drugs and 1596 proteins as their targets listed in the Drugbank database. In this graph, a link between a drug and protein signifies that the drug is active on that protein. An interaction between a pair of drugs indicates their association or connection based on whether or not they have a common target. For further analysis of measurements of similarity and variation in activity between drugs, we use information on activity of drugs on proteins to construct a weighted adjacency matrix. Every element of this matrix gives the pairwise weight measures of the interactions. An interaction is quantified in terms of the number of common targets (*s*) or the number of proteins on which the activity of two drugs differs (*d*). Thus both *s* and *d* are proportional to the Jaccard index used for calculation of chemical or structural similarity *c*. “Data: drugs, proteins, chemical similarity” section describes the data on drugs, proteins, activity, *c* used for this analysis.

### Interactions in the pharmacological topography: from cliffs to canyons

In this paper, an interaction between two drugs refers to the difference or similarity of activity of the drugs on the same protein. It is attributed a weight equal to the number of common or uncommon targets of the drugs. The discontinuities arising in the activity landscape due to sharp drops in similar activity along the chemical space represent drug interactions that are distinctive in nature [3, 4, 10–12]. They are not regular as they do not conform to the similarity principle. In other words, an interaction is distinctive when structurally similar drugs tend to behave oppositely on the same proteins. There may be two alternative specifications of a distinctive interaction in any pharmacological space. One, a pair of structurally similar drugs could have low similarity in activity. Two, the activity profiles of two structurally similar drugs may be highly dissimilar or variant. These are used alternatively for characterizing the well known phenomenon of activity cliff [3, 4, 10–12]. However, as we show here, this choice of measurement becomes crucial if *s* is measured by only the commonly active targets of drugs and ignores the commonly unresponsive proteins. Thus, the activity variation may not totally indicate the magnitude of similarity of drugs. We determine which of these behaviors is dominant in the present interaction space using the measures of *s*, *d*. We study their patterns with change in chemical similarity, and the implications for distinctiveness.

#### Similarity and variation

The weighted adjacency matrices *S* and *D* of drug–drug interactions measure the magnitude of similarity and difference or variation in activity of drug pairs respectively (“*S* and *D*” section). In the given pharmacological space, $0 \le S_{ij} \le 28; 0 \le D_{ij} \le 114$. These interaction weights representing elements of *S* and *D* are denoted by *s* and *d* respectively.

The measures applied facilitate the comparative analysis as they augment the differences in their magnitudes and ranges.

#### Comparison in the chemical space

**Fig. 1:**
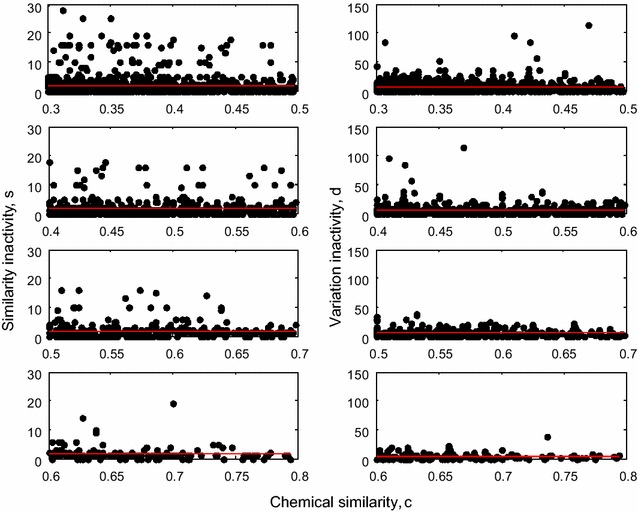
Pairwise weights of *s* (*left*) and *d* (*right*) with *c* in four overlapping ranges of *c* (*topmost* to *bottom* in order) of [0.3, 0.5], [0.4, 0.6], [0.5, 0.7], [0.6, 0.8]. The *red lines* in each subfigure represent the corresponding averages of *s* and *d* in the respective ranges of *c*. The units of *s* and *d* are given as the number of common and uncommon targets respectively. $0 \le s \le 28, 0 \le d \le 114$. Chemical similarity, *c* is given as $0 \le c \le 1$

We examine how the measures vary on all pairs of drugs in different ranges of chemical similarity, *c*. Figure 1 illustrates the comparison of *s* and *d* for the structure–activity association in progressing windows of *c*. Detailed inspection suggests that although the averages of both *s* and *d* are more or less constant over the chemical space, in every window, the fractions of the weights of *d* found above average remain higher than fractions of weights of *s* found above average. These *d* weights tend to be higher in magnitude than *s* weights owing to constructions of *s*, *d*. While both *s* and *d* decline with *c* overall, it must be noted that the decrease in *s* occurs rather gradually and hence the cliff representation may be deficient. Further, we compare the density of magnitudes (or number of points) found above average for *s* and *d* across each row of plots in Fig. 1. In all the subfigures, and particularly the ones in the intermediate region of the chemical space $0.3 \le c \le 0.65$, it is clear that region marked by above average *d* is more abundantly occupied by points than the corresponding region for *s*. This implies that a pair of structurally similar drugs is more likely to possess highly variant than highly similar activity.

Intermittent spikes in activity variation occur in the intermediate range of chemical similarity $c \in \left[ {0.3, 0.65} \right]$ or $c \in \left[ {0.4, 0.65} \right]$ with a dense layer of points beneath. This feature can be used to reasonably delineate the structure versus activity graphs from the perspective of activity variation. Thus in terms of variation, the topography maybe characterized as activity *canyons* or *gorges* depending on the steepness. This also applies to *s* but less distinctly.

### Quantifying structure and activity

In general, medicinal chemistry has relied on quantification of structure activity to obtain a function *f* such that—change in physiological activity = *f* (change in structure) [3, 4]. Here we see that the nature of the function and hence the landscape characterization would depend on which of the measures (*s* or *d*) is considered for quantifying biological activity. We are interested in the two dimensional interactions relating structure (*c*) and activity (*s* or *d*). Measures like SALI have been applied to quantify activity cliffs [12]. However, high SALI could also arise from high *c* (and low *d*) or low *c* (and high *d*). The overall score can be misleading for identification of distinctive interactions with high *c and* high *d*. We therefore present a probabilistic analysis of the each of the measures *s* and *d* jointly with *c*. This informs us not only the significance of cliffs but also where (if at all) they are found in the chemical space. In the canyon representation above, intermittent rises in the measures characterize distinctive interactions. The main purpose of this representation is for finding the probability of occurrence of various levels of (extreme) deviations. In other words, for every pair of drugs, it quantifies: *how distinctive and how probable?*

#### Predicted probability

**Fig. 2:**
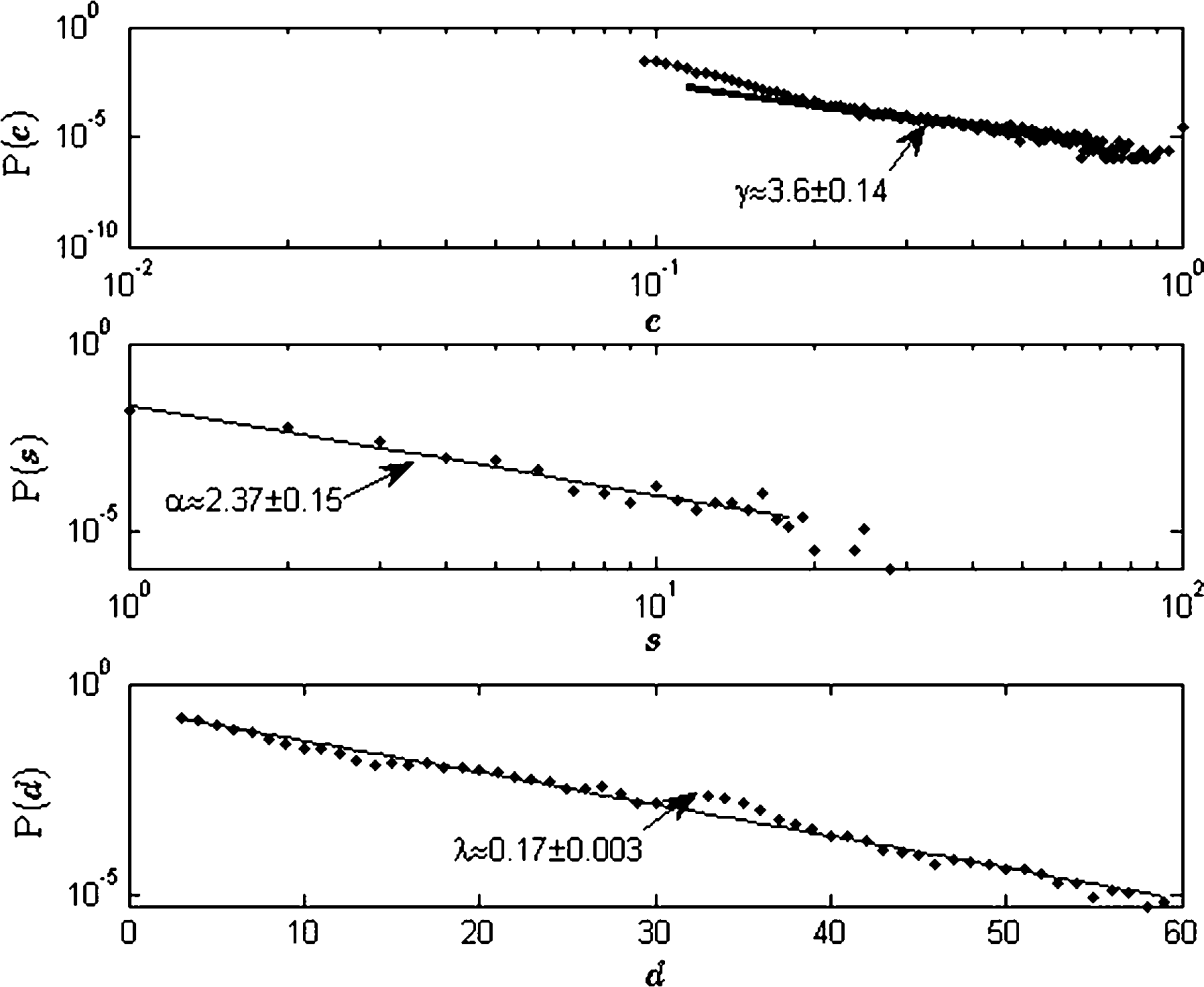
(*Top*) Probability of pairwise chemical or structural similarities P(C = *c*) on a log–log plot. The *straight line* approximates power law behavior on a range of *c* with exponent 3.6±0.14. (*Middle*) Probability of number of similar activities P(S = *s*) on log–log plot. The *straight line* approximates power law behavior on a range of *s* with exponent 2.37±0.15. (*Bottom*) Probability of number of dissimilar activities or variation P(D = *d*) on a semilog plot. The *straight line* approximates exponential behavior on a range of *d* with exponent 0.17±0.003

We compute joint probability of structural similarity with activity variation and with activity similarity, that is, the probabilities of (*c* and *s*) and (*c* and *d*). This analysis is based on the distributions of *c*, *s*, *d* weights representing all pairs of drugs. Figure 2 shows the different kinds of probability distributions of drug–drug interactions in terms of P(S = *s*), P(D = *d*), P(C = *c*). While *s* and *c* conform to a power law probability distribution, *d* follows an exponential distribution indicating that on the axis of *d*, the probability of high magnitudes diminishes greatly. This indicates that probability of finding extremely large variation (*d*) diminishes much more than the probability of finding very large *s* weights, however, the magnitudes of *d* are much higher than those of *s* when $c \ge 0.3$. Further, the power law exponents of *c*, *s* indicate that the variance of *c* is much higher than that of *s*, implying that *s* is more or less homogeneously distributed in the interaction space. “Probability distributions” section gives the mathematical forms and the estimation of the exponents (or constants). Note that the distributions found are approximations used to provide a generalized prediction of the propensity of distinctiveness.

In this interaction based predicted probability model, the joint probability $P\left( {s = s_{0} \cap c = c_{0} } \right)$ or $P\left( {d = d_{0} \cap c = c_{0} } \right)$ for each pair of drugs specifies the chance of finding the level of distinctiveness shown by the interaction between that pair of drugs. See “Joint probability computation” section for computation.

We find that the observed average (standard deviation) of *s* and *d* weights is 0.056 (0.5) and 7.44 (7.01) respectively. We compare the two measures by computing the predicted probabilities of *s* and *d* above their respective averages $P\left( {\left( {s \ge \mu_{s} } \right) \cap \left( {c_{1} \le c \le c_{2} } \right)} \right)$ and $P\left( {\left( {d \ge \mu_{d} } \right) \cap \left( {c_{1} \le c \le c_{2} } \right)} \right)$. Details of this procedure are given in “Joint probability computation” section. The probabilities calculated with the model for the categories *j* = 0, 1,…, 9 for *d* are almost consistently and significantly higher than those for *s*. When they are not higher, the difference is insignificant. We also compare the empirical probability of standardized scores of *s* and *d* being positive (above their averages). The probability of finding positive values of *d* is higher than that of *s*. It must be noted that *c* is considered at least equal to 0.3 because *c* < 0.3 is not significant for any analysis. Ignoring all pairs with *c* < 0.3 in this data, we have $\mu_{s} = 1.62$, $\sigma_{s} = 2.95$, $\mu_{d} = 4.8$, $\sigma_{d} = 7.53$.

##### Validation

1. We validate the predicted probability formulas (derived in “Joint probability computation” section) for the entire data of 1354 drugs by comparing it with the observed probability for categories $s \ge \mu_{s} + n\sigma_{s}$ and $d \ge \mu_{d} + n\sigma_{d}$ taking $c \ge 0.3$. For this purpose, we take observations and compute predictions at *n*= 0, 0.2, 0.4,…, 9. We use the averages and standard deviations for the entire (original) data and compute RMSE (see “Root mean square error” section) for judging the agreement between the predicted probability (*p*_*pred*_) and observed probability (*p*_*obs*_) for *s* and *d*. This is as low as 0.0001 and 0.0006 for calculations of *d* and *s* respectively. It is remarkable that while generalized predictions are made with the assumption of independence of *s* and *c*, and, *d* and *c* on all pairs of drugs, they confirm the observed dominance of above average *d* values. Figure 3 shows the agreement between observed and predicted values for *d*.

**Fig. 3:**
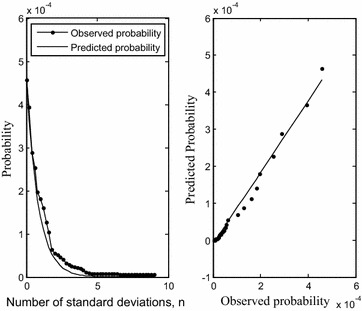
Observed and predicted probabilities (scaled) for $d \ge \mu_{d} + n\sigma_{d}$ and $c \ge 0.3$ plotted with *n* using the original data (*left*). Observed versus predicted probabilities showing a good agreement (*right*)
2. We now validate the predictions of the probability of distinctiveness using the system of drugs of the nervous system. This system consists of data on activity of 146 drugs on 219 protein targets. The probability distributions of interaction weights measured using pairwise *s*, *d*, *c* values for this system are similar to those shown in Fig. 2 but the patterns are not identical. While the probability distribution of *c* is the same as that obtained above, the distributions of *s* and *d* show approximately similar patterns as there may be some deviations. As before, we compare the observed probabilities with the predicted probabilities using the formulas, for categories $s \ge \mu_{s} + n\sigma_{s}$, $d \ge \mu_{d} + n\sigma_{d}$ taking $c \ge 0.3$. Using the information for this drug ensemble $c_{min} = 0.0089$, $s_{min} = 1, d_{min} = 0, \mu_{s} = 1.18, \sigma_{s} = 3.63,\mu_{d} = 16.67, \sigma_{d} = 13.39$, we show in Fig. 4 the agreement between observations and predictions for *d*. RMSE is 0.002 and 0.0006 for *s* and *d* calculations respectively. It is crucial to note that there is good agreement between observations and predictions despite the deviations from the assumptions and conditions involved in the construction of the formulas.

**Fig. 4:**
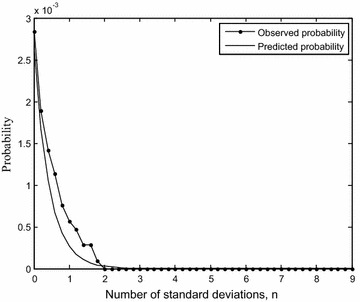
Observed and predicted probabilities (scaled) for $d \ge \mu_{d} + n\sigma_{d}$ and $c \ge 0.3$ plotted with *n* for the drugs of the nervous system

#### Hypotheses testing

We establish whether or not variation in activity dominates similarity in activity by comparing observed probabilities ($p_{1} , p_{2}$) in regions spanning the chemical space $0.3 \le c \le 1$. The tests are listed below. Tests 1–3 show the comparison for the region $0.3 \le c \le 1$. Comparisons between different regions are given in tests 4–5. Test 6 gives the significance of variation as a refined filter of rare distinctive interactions. “McNemar’s test for comparing proportions of *s* and *d* in the same chemical space” and “Paired t test” sections describe the two statistical tests applied. Reported below are the test results along with the significance level $\rho$,

1. $$p_{1} = P\left( {c \ge 0.3 \cap s \ge \mu_{s} } \right),\quad p_{2} = P\left( {c \ge 0.3 \cap d \ge \mu_{d} } \right)\quad p_{2} > p_{1} ,\;\rho < 0.01$$
2. $$p_{1} = P\left( {0.3 \le c \le 0.65 \cap s \ge \mu_{s} } \right),\quad p_{2} = P\left( {0.3 \le c \le 0.65 \cap d \ge \mu_{d} } \right)\quad p_{2} > p_{1} ,\;\rho < 0.01$$
3. $$p_{1} = P\left( {c \ge 0.65 \cap s \ge \mu_{s} } \right),\quad p_{2} = P\left( {c \ge 0.65 \cap d \ge \mu_{d} } \right)\quad p_{2} > p_{1} ,\;\rho \approx 0.1$$
4. $$p_{1} = P\left( {0.3 \le c \le 0.65 \cap s \ge \mu_{s} } \right),\quad p_{2} = P\left( {c \ge 0.65 \cap s \ge \mu_{s} } \right)\quad p_{1} > p_{2} ,\;\rho < 0.05$$
5. $$p_{1} = P\left( {0.3 \le c \le 0.65 \cap d \ge \mu_{d} } \right),\quad p_{2} = P\left( {c \ge 0.65 \cap d \ge \mu_{d} } \right)\quad p_{1} > p_{2} ,\;\rho < 0.05$$
6. $$p_{1} = P\left( {c \ge 0.65 \cap s \le \mu_{s} } \right),\quad p_{2} = P\left( {c \ge 0.65 \cap d \ge \mu_{d} } \right),\quad p_{1} > p_{2} ,\;\rho < 0.01.$$

**Fig. 5:**
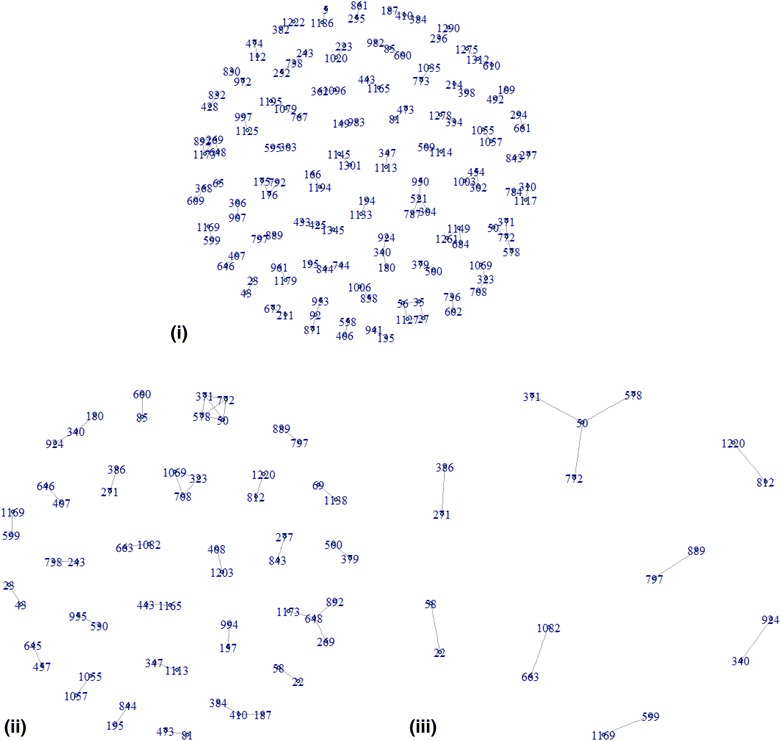
**i** Network of 91 distinctive interactions determined by *s* below average. **ii** Network of 39 distinctive interactions determined by *d* above average. **iii** Network of 11 distinctive interactions determined by *d* by calculation for $d \ge \mu_{d} + \sigma_{d} .$ The medicine pairs are (Lysine–Ornithone), (Adenosine monophosphate–Adenosine triphosphate), (Vitamin A–Alitretinoin), (Vitamin A–Tretinoin), (Vitamin A–Isotretinoin), (Propoiomazine–Aceprometazine), (Loxapine–Amoxapine), (Dicloxacillin–Cloxacillin), (Pseudoephidrine–Ephedrine), (Tioconazole–Miconazole), (Felodipine–Clevidipine)

Results of the first three tests confirm that variant activity behavior dominates the similar activity behavior of the drug pairs consistently in the chemical space. The probability of finding above average *d* magnitudes is more than that of finding above average *s* magnitudes in the region $c \ge 0.65$, but the difference is less significant. The intermediate region of the chemical space $0.3 \le c \le 0.65$ marks the transitional regime when structural similarity starts being positively associated with *d* more than with *s*. Distinctiveness emerges here. Tests 4, 5 indicate that *d* and *s* decrease with increase in *c*. However, *d* significantly dominates *s* in both proportion and magnitude particularly in the intermediate chemical region. We apply paired t test (“Paired t test” section) to compare these probabilities as they specify the effect of changing the level or range of *c* of interactions on their *s* and *d* weights. Test 6 establishes the advantage of *d* as a potential filter of distinctive interactions. Few above average *d* weights (32.5%) are higher in magnitude than 75% of below average *s* weights (Fig. 5i–iii). We identify all the rarely occurring distinctive interactions, most of which are not revealed with *s* but are crucial for characterization of activity landscape. Further, *d* yields distinctiveness at multiple levels of hierarchy set by criterion of the number of standard deviations above the average.

**Fig. 6:**
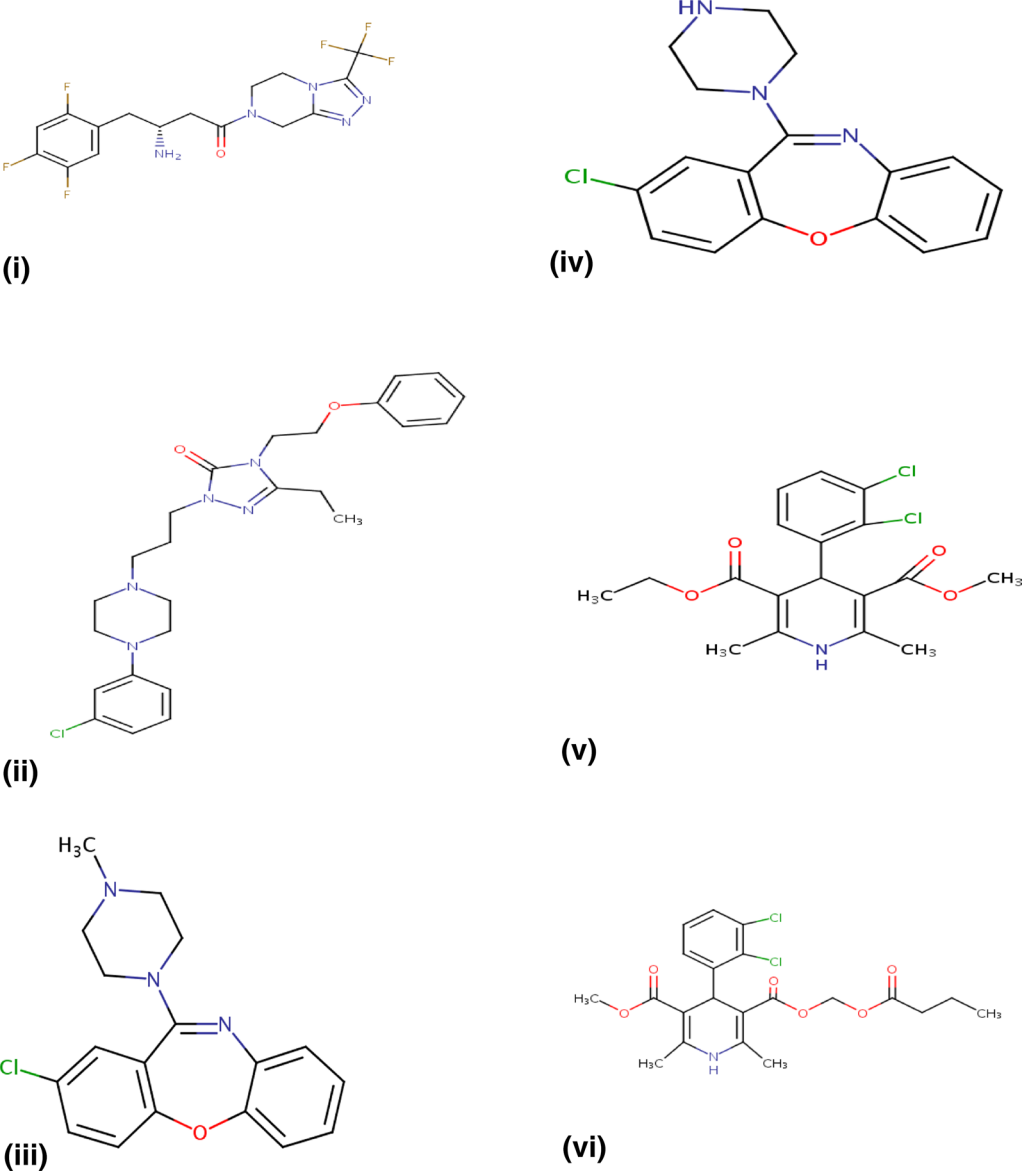
**i** Sitagliptin—used for control of type 2 diabetes mellitus, increased release of insulin. **ii** Nefazodone—analogous to Sitagliptin but affects the nervous system, has a palliative action. **iii** Loxapine-antipsychotic drug. **iv** Amoxapine—analogous to Loxapine but used for many other neurotic disorders and sedation. **v** Feldopine—calcium channel blocker for moderate hypertension. **vi** Clevidipine—analogous to Feldopine, is calcium channel blocker, but for advanced treatment of blood pressure

Most distinctive interactions occur between vitamins and medicines curing ailments of classes such as central nervous system or dermatological in nature. Several less similar interactions filtered as distinctive, include compound pairs from distinct classes. Figure 6i, ii shows structural similarity of Sitagliptin and Nefazodane that affect insulin release and central nervous system, respectively. Interestingly, highly variant interactions are fewer and more distinctive as they include drugs and their structural analogs belonging to the same class but performing intricately different functions. Figure 6iii–vi shows structurally similar Loxapine and Amoxapine affect the nervous system but Amoxapine is more versatile. Felodipine and Clevidipine are analogs for curing hypertension but Clevidipine treatment is more advanced. Thus, activity variation calculations are advantageous for identifying medicinal analogs that provide more specialized treatments for same kinds of ailments. We find that activities of such medicinal analogs typically vary a lot.

Tests 1–3 and 6 use McNemar test (“McNemar’s test for comparing proportions of *s* and *d* in the same chemical space” section). Test 6 compares *d* as a filter for highly distinctive drug interactions with *s*. We compare the probabilities $p_{1} = P\left( {c \ge 0.65 \cap s \le \mu_{s} - n\sigma_{s} } \right)$ and $p_{2} = P\left( {c \ge 0.65 \cap d \ge \mu_{d} + n\sigma_{d} } \right)$, *n* = 0, 1, 2…. For *n*=1, $p_{1} = 0$ and $p_{2} = \frac{11}{120} = 0.09.$ The *d* measure yields maximally distinctive interactions as *n* is increased, implying distinctiveness at multiple levels. Figure 5iii indicates this and for *n*=1 we report the pairs of drugs that are most distinctive. The proportion and nature of interactions filtered are significantly different from that obtained with *s*.

## Conclusions

This paper makes two contributions mainly. One is a methodological contribution as we provide a mathematical basis for identifying distinctive interactions, commonly studied as activity cliffs in literature. The predicted probability analysis we have introduced is based on simple assumptions but predicts the overall distinctiveness in various chemical regions to a reasonable accuracy. Further, it is able to predict the distinctiveness observed in a pharmacological space that may not be identical to the one considered for its construction. The second contribution is that this paper provides a new characterization of the pharmacological topography in the form of canyons. It is associated with the probabilistic analysis of distinctiveness and helps to filter the drug interactions at varying levels of distinctiveness and non-distinctiveness. This representation is appropriate for a probabilistic analysis because we use two measures—*s* and *d* that vary on highly different scales in the data. It identifies distinctive interactions and the region of the chemical space to find them. This methodology aims to rigorously quantify distinctiveness which can occur in diverse forms. This is particularly helpful as the presence of these deviations is known to hinder the quantitative structure activity modeling. Moreover, when similarity of activity is considered as given by the commonly active targets, some medicinal pairs may show both distinctiveness and non-distinctiveness and the cliff representation is not sufficient.

Activity cliffs have been studied in detail in past research. These aberrations make it harder to coherently generalize the quantitative association between changes in physiological activity and structure, which is vital for medicinal chemists. Methods applied can impact the decision process of preparing fresh drugs and drug development through identification of distinctive pairs.

The interpretation of the topography of pharmacological interaction space can vary according to the choice of measurement of *c*, *s*, *d*. We illustrate this with a probabilistic analysis of the pharmacological space which considers the probability of each of the measures *s* and *d* jointly with *c*. The consistency between observations and predictions corroborates that structural (*c*) and biological (*s* or *d*) properties can be considered as independently generated for studying the discontinuities in the landscape. A distinctive interaction can be measured either as high *c*, *d* or high *c* and low *s*. The almost contrasting construction of both measures results in a much higher range and distribution of magnitudes of *d* than of *s*. This affects the quantitative assessment of the interaction landscape, particularly for distinguishing structure–activity functions displayed by drugs. We establish using probability distributions of interaction attributes that it is less significantly likely for a pair of drugs with high structural similarity to act similarly than oppositely on a target. Further, the decrease in similar activity along the chemical space lacks the steepness of a cliff. In the given pharmacological space, high *d* dominates the chemical space in terms of not only magnitudes but also the probabilities. This behavior is mostly observed in the intermediate range of *c*, which we refer to as a *transitional* regime as it marks the increasing significance of *d*.

As *c* increases, interactions corresponding to high *d* are relatively rare and more distinctive than those of low *s*. This facilitates preparation of analogs for intricately specified treatments. Low *s* interactions can occur between drugs affecting different classes. However, distinctive interactions of high *d* are rarer and occur between structural analogs aimed to treat same kinds of ailments with increasingly intricate specifications. Such analogs providing advanced or versatile treatments of same kinds of ailments vary highly in their activity profiles. It is important to note that such associations are identified with high activity variation instead of low similarity.

Given the complex nature of structure–activity relationship study, a perfect conceptualization of the pharmacological topography may be hard. This analysis aims to suggest an alternative characterization resulting from mathematical and statistical formulations. A crowded valley of magnitudes with intermittent spikes of variation in the chemical subspaces, may be characterized as activity canyons or gorges. In a way this can be an encouraging sign for diversity in drug development. For instance, if a minor change in structure gives rise to an analog, then the two may be active on different kinds of targets more often than not. The sizes of their activity profiles may also be quite different. The methods provide a basis for practitioners and pharmacologists to identify distinctive interactions and the region of chemical space that they are probable to be observed in.

## Methods

### Data: drugs, proteins, chemical similarity

We use categorical binary activity results of 1354 drugs on 1596 protein targets. The activity information is given as binary attributes, *A*_*i(k)*_ = 1 if a drug *i* is active on protein *k* and 0 if the drug is not active. This constitutes the activity profile of drug *i.* Chemical similarity of drugs is computed pairwise with tanimoto similarity method [17]. The weighted network of *N* = 1354 drugs, *C*, has $N \times N$ weighted adjacency matrix. The elements of the matrix, *C*_*ij*_ give the chemical similarity of the pair of drugs and $0 \le C_{ij} \le 1$.

### Pairwise similarity and variation in activity of drugs

#### *S* and *D*

***S***—If *A*_*i*_ and *A*_*j*_ are activity profiles of drugs *i,j*, that is the proteins *k* for which *A*_*i(k)*_ =1 and *A*_*j(k)*_ =1, then $S_{ij} = \left| {A_{i\left( k \right)} \cap A_{j\left( k \right)} } \right|.$ Here *S* has $N \times N$ weighted adjacency matrix. We denote the similarity weights or magnitudes as *s*. The commonly unresponsive proteins are ignored. Every element of the matrix denotes the weight of the connection or the interaction between pairs of drugs.

***D***—Variation between the activity profiles of two drugs measures the number of differences in their binary attributes, that is the number of proteins on which they differ in activity. The weighted network *D* of pairwise distances of drugs $N \times N$ weighted adjacency matrix $D_{ij} = \left| {\left( {A_{i\left( k \right)} \cap \overline{{A_{j\left( k \right)} }} } \right) \cup \left( {\overline{{A_{i\left( k \right)} }} \cap A_{j\left( k \right)} } \right)} \right|$. This is almost complement of *S* as we ignore the commonly unresponsive proteins on all pairs of drugs while computing *S*. Both these measures [15, 16] are constructed along the lines of Jaccard similarity and distance coefficient without normalization. We’re interested in constructions of *S* and *D* so as to make their magnitudes and variances distinct, hence we compare the actual numbers of similar and variant activities.

### Predicted probability model

#### Probability distributions

Assuming continuous distributions for the pairwise weights as random variables for similarity (*s*), variation (*d*), chemical similarity (*c*), we write the mathematical forms of the normalized probability distributions as

$$P\left( s \right) = \frac{0.045}{{s_{min} }} \left( {\frac{s}{{s_{min} }}} \right)^{ - \alpha } ;$$

$$P\left( d \right) = 0.042 e^{{\lambda d_{min} }} e^{ - \lambda d} ;$$

$$P\left( c \right) = \frac{\gamma - 1}{{c_{min} }} \left( {\frac{c}{{c_{min} }}} \right)^{ - \gamma } .$$

$P\left( s \right), P\left( c \right)$ are power law distributions and $P\left( d \right)$ is exponential. The behaviors are shown in Fig. 2. The constants $\alpha , \gamma , \lambda$ are computed as regression coefficients given as slopes of the straight lines in the 3 plots shown in Fig. 2 with probability *p* shown on the vertical axis. The first two top figures for *c* and *s* are on a log–log scale, so that the straight lines are given by $\log \left( {p\left( s \right)} \right) = \alpha \log \left( s \right) + intercept$ and $\log \left( {p\left( c \right)} \right) = \gamma \log \left( c \right) + intercept$. The straight line in Fig. 2 (bottom) is on a semi-log scale, given as $\log \left( {p\left( d \right)} \right) = \lambda d + intercept.$ Note that each of these regression equations implies that the probability in every case is proportional to $e^{intercept}$. This does not affect the calculation of the slope constants. Here *s*_*min*_*, c*_*min*_*, d*_*min*_ correspond to the minimum values of biological, structural similarity and variation respectively. We have *d*_*min*_ = 0 and for a well defined power-law distribution, we consider *s*_*min*_ = 1, *c*_*min*_ = 0.004.

#### Joint probability computation

The joint probabilities using *s* and *d* are $P\left( {s = s_{0} \cap c = c_{0} } \right)$ and $P\left( {d = d_{0} \cap c = c_{0} } \right)$ respectively. *s*_0_*, c*_0_*, d*_0_ are parameters denoting magnitudes of *s*, *c*, *d* which we vary. In the same way, we can specify ranges of study $s_{1} \le s \le s_{2}$, $c_{1} \le c \le c_{2}$, $d_{1} \le d \le d_{2}$ and find the corresponding probabilities.

*If structure and activity are independently generated, then predicted joint probabilities are given by*

$$P\left( {\left( {c_{1} \le c \le c_{2} } \right) \cap \left( {s_{1} \le s \le s_{2} } \right)} \right) = c_{min}^{\gamma - 1} s_{min}^{\alpha - 1} \left( {s_{1}^{1 - \alpha } - s_{2}^{1 - \alpha } } \right)\left( {c_{1}^{1 - \gamma } - c_{2}^{1 - \gamma } } \right)\;and$$

$$P\left( {\left( {c_{1} \le c \le c_{2} } \right) \cap \left( {d_{1} \le d \le d_{2} } \right)} \right) = c_{min}^{\gamma - 1} \frac{0.042}{\lambda }\left( {e^{{ - \lambda d_{1} }} - e^{{ - \lambda d_{2} }} } \right)\left( {c_{1}^{1 - \gamma } - c_{2}^{1 - \gamma } } \right)$$

*Proof* As structure and activity are independently generated, using probability distributions of *P*(*c*) and *P*(*s*) from above, the joint probabilities approximate to

1 $$P\left( {\left( {c_{1} \le c \le c_{2} } \right) \cap \left( {s_{1} \le s \le s_{2} } \right)} \right) = P\left( {c_{1} \le c \le c_{2} } \right)P\left( {s_{1} \le s \le s_{2} } \right) = \mathop \int \limits_{{s_{1} }}^{{s_{2} }} \frac{0.045}{{s_{min} }} \left( {\frac{{s^{{\prime }} }}{{s_{min} }}} \right)^{ - \alpha } ds'\mathop \int \limits_{{c_{1} }}^{{c_{2} }} \frac{\gamma - 1}{{c_{min} }} \left( {\frac{{c^{{\prime }} }}{{c_{min} }}} \right)^{ - \gamma } dc^{{\prime }}$$

Similarly, using probability distributions of *P*(*c*) and *P*(*d*), we obtain

2 $$P\left( {\left( {c_{1} \le c \le c_{2} } \right) \cap \left( {d_{1} \le d \le d_{2} } \right)} \right) = P\left( {c_{1} \le c \le c_{2} } \right)P\left( {d_{1} \le d \le d_{2} } \right) = \mathop \int \limits_{{d_{1} }}^{{d_{2} }} 0.042 e^{{\lambda d_{min} }} e^{{ - \lambda d^{\prime}}} dd^{{\prime }} \mathop \int \limits_{{c_{1} }}^{{c_{2} }} \frac{\gamma - 1}{{c_{min} }} \left( {\frac{{c^{{\prime }} }}{{c_{min} }}} \right)^{ - \gamma } dc^{{\prime }}$$

Solving Eqs. (1) and (2) we obtain

3 $$P\left( {\left( {c_{1} \le c \le c_{2} } \right) \cap \left( {s_{1} \le s \le s_{2} } \right)} \right) = c_{min}^{\gamma - 1} s_{min}^{\alpha - 1} \left( {s_{1}^{1 - \alpha } - s_{2}^{1 - \alpha } } \right)\left( {c_{1}^{1 - \gamma } - c_{2}^{1 - \gamma } } \right)$$

4 $$P\left( {\left( {c_{1} \le c \le c_{2} } \right) \cap \left( {d_{1} \le d \le d_{2} } \right)} \right) = c_{min}^{\gamma - 1} \frac{0.042}{\lambda }\left( {e^{{ - \lambda d_{1} }} - e^{{ - \lambda d_{2} }} } \right)\left( {c_{1}^{1 - \gamma } - c_{2}^{1 - \gamma } } \right)$$

Equations (3) and (4) give the predicted probabilities of simultaneously finding structural and activity similarity, and, structural similarity and activity variation in a range, respectively.

We compare the two measures by computing the predicted probabilities of similarity and distance above their respective averages. We denote the average and standard deviation (respectively) of *s* as $\mu_{s}$, $\sigma_{s}$ and of *d* as $\mu_{d}$, $\sigma_{d}$. This is given by comparing Eqs. (3) and (4) taking *s*_*min*_ = 1, *c*_*min*_ = 0.004, *d*_*min*_ = 0, $s_{1} = \mu_{s}$, $s_{2} = \infty$, $d_{1} = \mu_{d}$, $d_{2} = \infty$, in different ranges of [c1, c2] as [0.3,) [0.3, 0.65], [0.65,) for the purpose of integration. In the range of $c \ge 0.3$, we have $\mu_{s} = 1.62$, $\sigma_{s} = 2.95$, $\mu_{d} = 4.85$, $\sigma_{d} = 7.53$. It must be noted that we compute the values of these averages and standard deviations for all progressing windows along the chemical space.

In order to compare *s* with *d* above their respective averages, we measure the predicted probability of finding $s \ge \mu_{s} + n\sigma_{s}$ and $d \ge \mu_{d} + n\sigma_{d}$ for *n*=0,1, 2… respectively, in the same chemical space. The standard deviations may also be increased in steps of 0.2. These are considered as measurements for the categories $P\left( {\left( {s \ge \mu_{s} } \right) \cap \left( {c_{1} \le c \le c_{2} } \right)} \right)$ and $P\left( {\left( {d \ge \mu_{d} } \right) \cap \left( {c_{1} \le c \le c_{2} } \right)} \right)$ for similar and variant activities respectively. We also find the probabilities for standardized data where all the values of *s* and *d* are subtracted from their averages and divided by their standard deviations for a certain chemical space. Then the comparison is between the probability of finding *s* and *d* above their average = 0 in a given chemical space.

#### Root mean square error

$RMSE = \sqrt {\sum\nolimits_{j} {\frac{{(p_{pred} - p_{obs } )^{2} }}{j - 1}} } ,\quad j = 1,2 \ldots$ Note that we compute the observed values by considering the number of pairs sharing both properties of being within the given range of *c* and *s* (or *d*). Thus $P\left( {s \cap c} \right) = P\left( {s |c} \right)P\left( c \right).$ If we find the joint probability of the two properties in a particular range of *c* then *P*(*c*) = 1.

### Hypothesis testing

In order to test the significance of the difference between the observed probabilities associated with *s* and *d*, we apply two tests.

#### McNemar’s test for comparing proportions of *s* and *d* in the same chemical space

We use this test to compare the relative frequencies (or probabilities) of *s* and *d* above their averages in the same chemical region $c \in \left[ {c_{1} ,c_{2} } \right]$. This test is suitable for comparisons of the [18] two measurements made on the same pairs. The contingency table for a given chemical region is

**Table:** 

	$d \ge \mu_{d}$	$d < \mu_{d}$
$s \ge \mu_{s}$	$n_{1}$	$n_{2}$
$s < \mu_{s}$	$n_{3}$	$n_{4}$

If $n_{1} + n_{2} + n_{3} + n_{4} = n,$ we test whether or not marginal probabilities are equal: $\frac{{n_{1} + n_{2} }}{n} = \frac{{n_{1} + n_{3} }}{n}$ and $\frac{{n_{3} + n_{4} }}{n} = \frac{{n_{2} + n_{4} }}{n}.$ The null and alternative hypotheses can be written as

H_0_: $$n_{2} = n_{3}$$

H_1_: $n_{2} < n_{3}$ (frequency of $d \ge \mu_{d}$ is greater than that of $s \ge \mu_{s}$ in the given chemical region)

We reject H_0_ in favor of H_1_ at the level of significance $\rho$ if $\frac{{\left( {n_{2} - n_{3} } \right)^{2} }}{{n_{2} + n_{3} }} > \chi_{1}^{2}$, or if binomial probability $P(X \ge n_{3} |n = n_{2} + n_{3} , p = 0.5) \le \rho$. The comparisons made here suit the design of the analysis, hence no corrections are required. Further, $\rho$ of the tests is sufficiently low for the tests to be significant after correction.

#### Paired t test

This test is used to compare the average of the difference between probabilities $p_{1} = P\left( {\left( {c_{1} \le c \le c_{2} } \right){\bigcap }\left( {s \ge \mu_{s} + n\sigma_{s} } \right)} \right)$ and $p_{2} = P\left( {\left( {c_{1} \le c \le c_{2} } \right){\bigcap }\left( {d \ge \mu_{d} + n\sigma_{d} } \right)} \right)$ observed (pairwise) at *n* = 0, 1, … 8. We also sample the observations by increasing standard deviations in intervals of 0.2. All the probabilities so computed can be generalized as $p_{1} = P\left( {\left( {c_{1} \le c \le c_{2} } \right){\bigcap }\left( {s \ge \mu_{s} } \right)} \right)$ and $p_{2} = P\left( {\left( {c_{1} \le c \le c_{2} } \right){\bigcap }\left( {d \ge \mu_{d} } \right)} \right)$. These probabilities represent the results obtained by two different measures of interaction distinctiveness—*s*, *d*. In this way, we consider the variation of probabilities in the space above the average sampled at increasing number of standard deviations.

We test the null hypothesis H_0_ of equality of probabilities on average against one-sided alternative H_1_

H_0_: $$\Delta = 0$$

H_1_: $$\Delta < 0$$ or H_1_: $$\Delta > 0$$

with the test statistic, $t = \frac{\Delta }{{\sigma_{\Delta } /n}}$ at significance level $\rho = 0.05$ for *n* − 1 d.f. Here $\Delta$ represents the vector of differences, *p*_1_ − *p*_2_, $\sigma_{\Delta }$ is the standard deviation of $\Delta$ and sample size *n* = 9 here. The null hypothesis of no difference is rejected in favor of alternate H_1_ if the observed *t* is greater than the critical value of test statistic i.e. *t* > *t*_*c*_ (0.05,8). We apply this for comparing probabilities of *s* and *d* in different chemical regions.
